# Application of geospatial analysis for mapping the distribution of severe maternal morbidities in eastern Ethiopia: The case of the Ethiopian obstetric surveillance system

**DOI:** 10.1002/ijgo.70973

**Published:** 2026-04-07

**Authors:** Sisay Mulugeta Alemu, Gerd Weitkamp, Marian Knight, Sagni Girma, Mohammed Yuya, Jelle Stekelenburg, Thomas van den Akker, Regien Biesma, Abera Kenay Tura

**Affiliations:** ^1^ Global Health Unit, Department of Health Sciences University Medical Center Groningen Groningen the Netherlands; ^2^ Policy & Implementation Research for Cancer Prevention DKFZ Hector Cancer Institute at the University Medicine Mannheim Heidelberg Germany; ^3^ Department of Cultural Geography, Faculty of Spatial Sciences University of Groningen Groningen the Netherlands; ^4^ National Perinatal Epidemiology Unit University of Oxford Oxford UK; ^5^ School of Nursing and Midwifery College of Health and Medical Sciences, Haramaya University Haramaya Ethiopia; ^6^ Department of Obstetrics and Gynecology Leiden University Medical Center Leiden the Netherlands; ^7^ School of Public Health College of Health and Medical Science, Haramaya University Haramaya Ethiopia; ^8^ Department Obstetrics & Gynaecology Leeuwarden Medical Centre Leeuwarden the Netherlands; ^9^ Athena Institute Vrije Universiteit Amesterdam Amsterdam the Netherlands; ^10^ Julius Center Global Health University Medical Center Utrecht Utrecht the Netherlands; ^11^ Department of Obstetrics and Gynaecology University Medical Centre Groningen, University of Groningen Groningen the Netherlands; ^12^ Department of International Public Health Liverpool School of Tropical Medicine Liverpool UK

**Keywords:** Ethiopia, geospatial analysis, maternal complications, obstetric surveillance

## Abstract

**Introduction:**

The disparity in maternal mortality and severe morbidity between high‐ and low‐income countries is well established. Previously, we highlighted within‐country disparities in Ethiopia using demographic and health survey data. This study used enhanced obstetric surveillance data to detect subnational hotspot areas and factors associated with disparities in severe maternal morbidity in eastern Ethiopia.

**Methods:**

This study used data from the Ethiopian Obstetric Surveillance System (EthOSS) study, which collected data for all women who experienced severe maternal morbidity in 13 hospitals in eastern Ethiopia between April 2021 and March 2022. Women whose geographical location was not recorded were excluded. We used optimized hotspot analysis to identify areas with higher rates of severe maternal morbidity while controlling for population density and conducted linear and geographically weighted regression analyses to assess factors associated with the distribution.

**Results:**

Of all 2043 women with severe maternal outcomes, 1775 (87%) women with severe maternal morbidity with complete geographical information were included for analysis. Less than half (47%) lived within the recommended 2‐h travel time to the nearest emergency obstetric and newborn care (EmONC) facility, with significant geographic variation. Hotspot analysis identified clusters of high rates near urban centers such as Dire Dawa and Harari even after controlling for the population density, while lower rates were found in eastern Oromia. Geographically weighted regression analysis showed that proximity to health facilities, especially to a basic or comprehensive EmONC facility, was associated with higher maternal complication rates.

**Conclusion:**

This study highlights the value of leveraging geocoded surveillance data to conduct geospatial analyses to uncover spatial patterns. We found a higher rate of severe maternal morbidity in the larger cities, indicating that the urban population had better access to care during obstetric complications, while rural and remote areas with limited access might fail to come to hospitals when complications arise or die at home or lower‐level facilities.

## 1 INTRODUCTION

Maternal health remains a critical global concern, especially in low‐ and middle‐income countries, where maternal mortality rates are unacceptably high. In Ethiopia, the maternal mortality ratio is estimated to be 267 deaths per 100 000 live births.^1^ For every maternal death, an estimated 20–30 women have severe maternal morbidity, also known as maternal near‐miss.^2^, ^3^ A severe maternal morbidity is a life‐threatening complication that occurs during pregnancy, childbirth, or within 42 days of termination of pregnancy and nearly results in a woman's death but from which she survives.^4^, ^5^ Despite some improvements in Ethiopia's maternal mortality ratio, the burden of severe maternal morbidity remains a significant challenge.^6^ The prevalence ranges from 12.6% to 14% in Ethiopia.^7^, ^8^ In addition, complications during pregnancy can have long‐lasting consequences, including chronic health conditions that profoundly affect a woman's well‐being and her ability to fulfill her roles and responsibilities within her family and community.^6^, ^9^, ^10^ Therefore, addressing severe maternal morbidity is also crucial, alongside the primary focus on maternal mortality, for improving women's overall health.

In countries such as Ethiopia, with severe under‐reporting of maternal deaths, studying severe maternal morbidity is essential to monitor the quality of obstetric care and for taking corrective actions. Studying severe maternal morbidity offers several advantages over focusing solely on maternal deaths. It increases the number of cases for analysis, as severe maternal morbidity is more common than mortality. Additionally, it is less threatening to clinicians and, therefore, less likely to be under‐reported. Further, unlike maternal death studies, analyzing severe maternal morbidity can capture the voices of women who share similar characteristics with those who suffer maternal mortality.^3^

Geospatial analysis, the process of collecting, analyzing, and interpreting geographic data, plays a crucial role in understanding the spatial distribution of health services, disease patterns, and risk factors. This analysis is a powerful tool for improving decision‐making in maternal and newborn health by identifying geographical inequalities, providing detailed insights, and helping to target intervention programs in areas where maternal deaths and morbidities are most likely to occur.^11^ Data collection for geospatial analysis often requires Global Positioning System (GPS) technology to gather precise geographic coordinates of health facilities, communities, and households or remote sensing and satellite data. As a result, many geospatial studies on maternal or neonatal health in sub‐Saharan Africa use data from large surveys, such as Demographic and Health Surveys and Service Provision Assessment surveys,^12^ which collect location information using GPS.^13^ However, such large‐scale surveys can be costly and conducted infrequently and might not always cover particular health issues, such as severe maternal morbidity. In Ethiopia, smaller surveys on a broader range of health topics are available, although they often collect only basic geographic information, such as the name of the administrative area where participants live. The Ethiopian Obstetric Surveillance System (EthOSS) is one such survey, which collects data on maternal deaths and severe maternal morbidity in eastern Ethiopia.^14^

Although several studies on severe maternal morbidity exist in Ethiopia, the primary focus is on estimating the national and/or regional prevalence and exploring associated factors^7^, ^8^, ^15^, ^16^, ^17^, ^18^, ^19^; the geographic distribution of severe maternal morbidity has not been studied. Our previous study identified that maternal mortality is unevenly distributed in Ethiopia, with high mortality observed in the central Amhara and Oromia regions and low mortality in eastern Ethiopia.^20^ Identifying hotspot areas of severe maternal morbidities might inform the deployment of targeted interventions to address disparities in maternal health outcomes and prevent maternal deaths. This research aimed to identify regional hotspot areas of severe maternal morbidity in eastern Ethiopia and factors associated with disparities using the Ethiopian Obstetric Surveillance System data.

## 2 METHODS

This retrospective, institution‐based observational study using prospectively collected surveillance data from April 2021 to March 2022 across 13 hospitals in eastern Ethiopia, including eight general hospitals, three primary hospitals, one referral hospital, and one tertiary hospital. The study workflow is summarized Figure 1.

**FIGURE 1:**
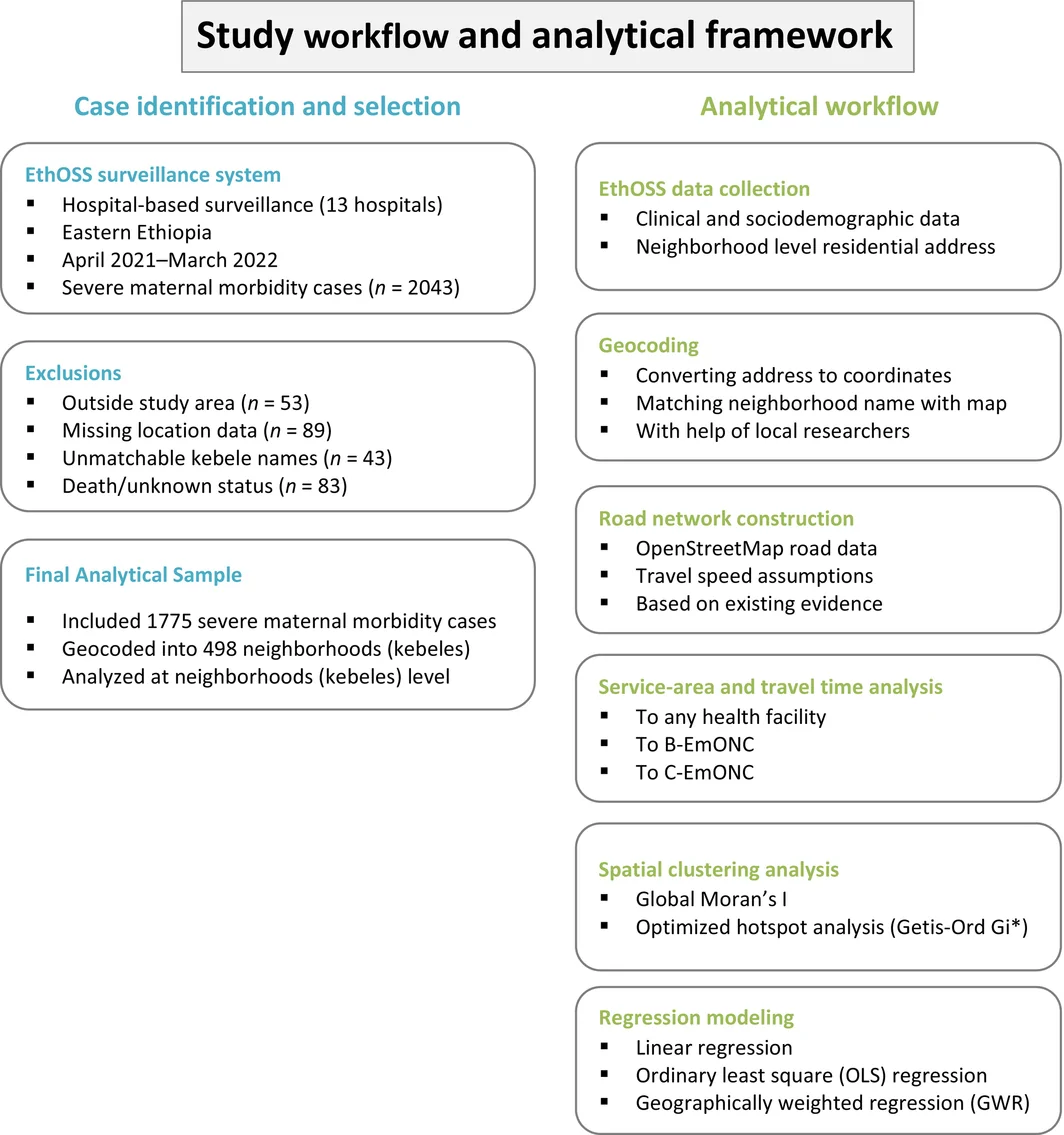
Study workflow and analytical framework.

### 2.1 Population and data source

This study utilized data from the EthOSS survey, a collaborative project between Haramaya University, the University of Oxford, Leiden University Medical Centre, and the University Medical Center Groningen, conducted in 13 hospitals in eastern Ethiopia,^14^, ^21^ which includes the Harari Region, Dire Dawa City Administration, and the East and West Hararghe Zones of the Oromia Region (Figure 2). The participating hospitals ranged from rural district hospitals to urban tertiary hospitals. All women who experienced severe maternal morbidity, such as obstetric hemorrhage, eclampsia, uterine rupture, sepsis, and severe anemia, during pregnancy, childbirth, or within 42 days of pregnancy termination and visited the 13 hospitals from April 2021 to March 2022 were included in the study.

**FIGURE 2:**
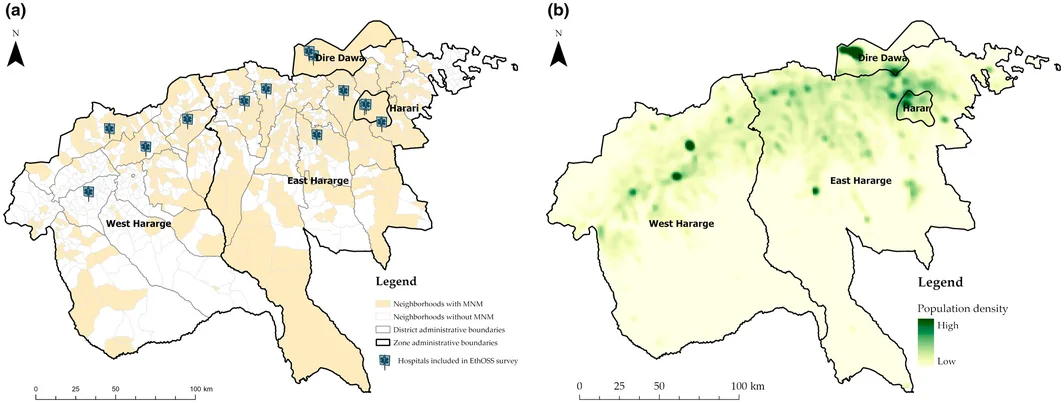
Kebeles (neighborhoods) with and without severe maternal morbidity in eastern Ethiopia, as well as the locations of hospitals that participated in the Ethiopian Obstetric Surveillance System (EthOSS) survey (left) and population density of women of reproductive age (15–49) in eastern Ethiopia (right).

We used publicly available population distribution data for women of reproductive age (15–49 years) from Facebook Connectivity Lab and the Center for International Earth Science Information Network, Columbia University.^22^ These data provide 2019 estimates of the number of reproductive‐age women at 30 × 30‐m spatial resolution.

### 2.2 Variable and measurement

The primary outcome variable for this study was severe maternal morbidities (obstetric hemorrhage, eclampsia, uterine rupture, sepsis, or severe anemia) during pregnancy, childbirth, or within 42 days postpartum. All women with the above obstetric conditions were included as a severe maternal morbidity case. The detailed working definitions of obstetric conditions included in EthOSS can be found elsewhere.^14^ Sociodemographic, clinical, and geographic information was collected by reviewing the medical records. Due to logistical constraints, the precise geographic coordinates of the women's households were not initially collected. Instead, location information was collected at the smallest administrative unit known as “kebele.” Kebele is the smallest administrative unit in Ethiopia; with a population size of 3000–5000, it is equivalent to and hereafter referred to as the neighborhood.

### 2.3 Data preparation

Geocoding, a technique for converting place names to map coordinates, was used to locate the neighborhood where the women lived. We matched neighborhood name reported by participants with administrative neighborhood names of Ethiopia GeoPortal datasets.^23^ For women whose neighborhood names could not be matched, often due to misspellings or presence of multiple names for a single neighborhood, we attempted to match their addresses by referring to other contextual clues, such as the district and village (if reported) they lived in and the health facility they were referred from. This process was carried out iteratively with the assistance of two researchers with extensive knowledge of the study area (SG, MY) until the team agreed the address was unmatchable. Women whose residential addresses were unavailable or could not be matched to the list of kebeles in the locational data were excluded from the analysis, as precise geospatial coordinates were required for the study.

### 2.4 Statistical analysis

The analyses were conducted at the neighborhood level for all women experiencing severe maternal morbidity who presented to participating hospitals during the study period, and no group assignment occurred. We built road networks using OpenStreetMap road data.^24^ We assumed that women would walk to the nearest available road at 2 km/h and use public transport afterward with average speeds of 80, 50, 20, 10, and 5 km/h for highway, primary, secondary, tertiary, and other roads. The speed assumptions were based on the findings of a systematic review of geospatial studies in sub‐Saharan Africa.^25^ The detailed assumptions and procedures used for building a road network can be found elsewhere.^26^ We conducted a Service Area Network analysis to estimate travel time from each neighborhood to emergency obstetric and newborn care (EmONC) facilities. An EmONC facility is a health facility capable of providing essential interventions to prevent and manage complications, including basic EmONC (B‐EmONC) facilities, which provide basic life‐saving services, and C‐EmONC facilities, which offer additional life‐saving interventions such as cesarean sections and blood transfusions.^27^, ^28^

We also performed spatial autocorrelation analysis using Global Moran's I in ArcGIS Pro (version 3.4) to assess the clustering pattern of severe maternal morbidity rates. Subsequently, we conducted an optimized hotspot analysis to identify statistically significant geographic clusters, hotspots, and cold spots of severe maternal morbidity rates across the study area using women's residential addresses. The Getis‐Ord Gi* statistic was used to pinpoint the location and intensity of these hotspot areas. To account for population distribution, we used the severe maternal morbidity rate, the ratio of severe maternal morbidities to the total population in each neighborhood, ensuring the hotspot analysis would not be skewed by high population density areas. The model selected an optimal fixed distance band of 16.9 km based on neighborhood spatial configuration. The null hypothesis in the Getis‐Ord Gi* analysis is that morbidity is uniformly distributed across the study area. Therefore, hotspot areas are neighborhoods with statistically significantly higher morbidity rates than expected if the morbidity is uniformly distributed, while cold spot areas have significantly lower morbidity rates than expected.

We conducted a linear regression analysis to identify factors associated with severe maternal morbidities at the neighborhood level. We included all relevant clinical and demographic variables measured in the EthOSS survey that we believed could be associated with severe maternal morbidity. Variables were selected based on theoretical and clinical relevance, informed by established literature on severe maternal morbidity.^5^, ^29^ Distance‐related variables, that is, the distance of each neighborhood to health facilities and the type of facility closest to each neighborhood, were calculated using network analysis and incorporated into the model. Marital status was excluded due to a large number of missing observations. All variables were entered into the model simultaneously using the “Enter” method. Further, we performed ordinary least squares regression in ArcGIS Pro and found that the residuals were spatially autocorrelated, indicating a spatially varying relationship. Finally, we carried out a geographically weighted regression using ArcGIS Pro. Because the severe maternal morbidity rate was not normally distributed, we performed a logarithmic transformation of the rate before using it as the outcome variable in the regression analysis. The scale of analysis was determined using a distance band selected by the model through a golden search approach. For all regression analyses, neighborhoods with no reported cases of severe maternal morbidity were excluded due to the absence of corresponding explanatory variable data, as no women from these areas participated in the EthOSS survey.

## 3 RESULTS

### 3.1 Descriptive

Of a total of 2043 women with severe maternal outcomes identified in the EthOSS survey, 1775 women with severe maternal morbidity with a mean age of 25 years, whose geocoding was successful, were included; 53 were excluded because they came from outside the study area; 89 had missing location information; 43 reported location details that could not be matched with existing administrative kebeles; and 83 had died, or their status was unknown. Most complications (85%) occurred after giving birth (Table 2). The 1775 women included in this study came from 498 neighborhoods, accounting for approximately half (51%) of the total 970 neighborhoods in eastern Ethiopia (Figure 2).

### 3.2 Service area

The median travel time from each neighborhood to the nearest health facility was 48 min, while the median travel time to the closest EmONC facility (i.e., a B‐EmONC or C‐EmONC facility) was 94 min. Notably, the median travel time to the closest C‐EmONC facility was 211 min (Figure 3 and Table 2).

**FIGURE 3:**
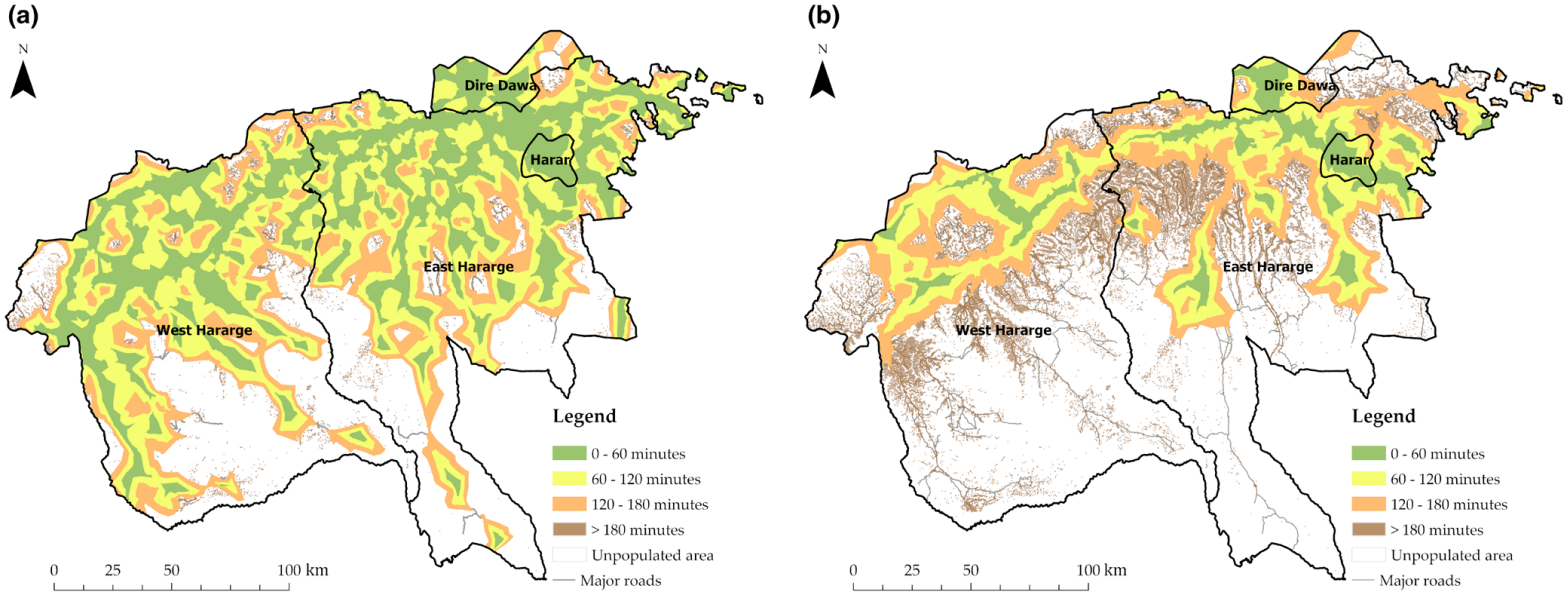
Distance of different areas in eastern Ethiopia from the nearest emergency obstetric and newborn care facilities (left) and any health facility (right).

Less than half of women live within the recommended 2‐h travel time to the nearest basic or comprehensive EmONC facility. Notably, this access varies significantly across the region. While almost all women in Harar live within the recommended 2‐h travel time, only one‐third of women in West Hararghe have such access (Table 1).

**TABLE 1:** Percentage of the eastern Ethiopian population within a 2‐h travel distance of emergency obstetric and newborn care facilities.

	Total population	Number of population within 2 h of EmONC	Percentage of population within 2 h of EmONC (%)
Harar	63 190	60 832	96
Dire Dawa	125 994	88 496	70
East Hararghe	811 542	387 619	48
West Hararghe	538 760	189 233	35
Total	1 539 485	726 181	47

Abbreviation: EmONC, emergency obstetric and newborn care.

### 3.3 Hotspot analysis

We found that severe maternal morbidity rates were not uniformly distributed across the study area, as evidenced by a statistically significant spatial autocorrelation analysis (*P* < 0.001). Similarly, optimized hotspot analysis identified statistically significant geographic clusters of high rates, concentrated mainly around the larger cities of Dire Dawa and Harar. In contrast, cold spot areas, with lower than expected severe maternal morbidity rates, were found in the eastern parts of the Oromia region, near the border with the Somali region, and in the central parts of West Hararghe (Figure 4).

**FIGURE 4:**
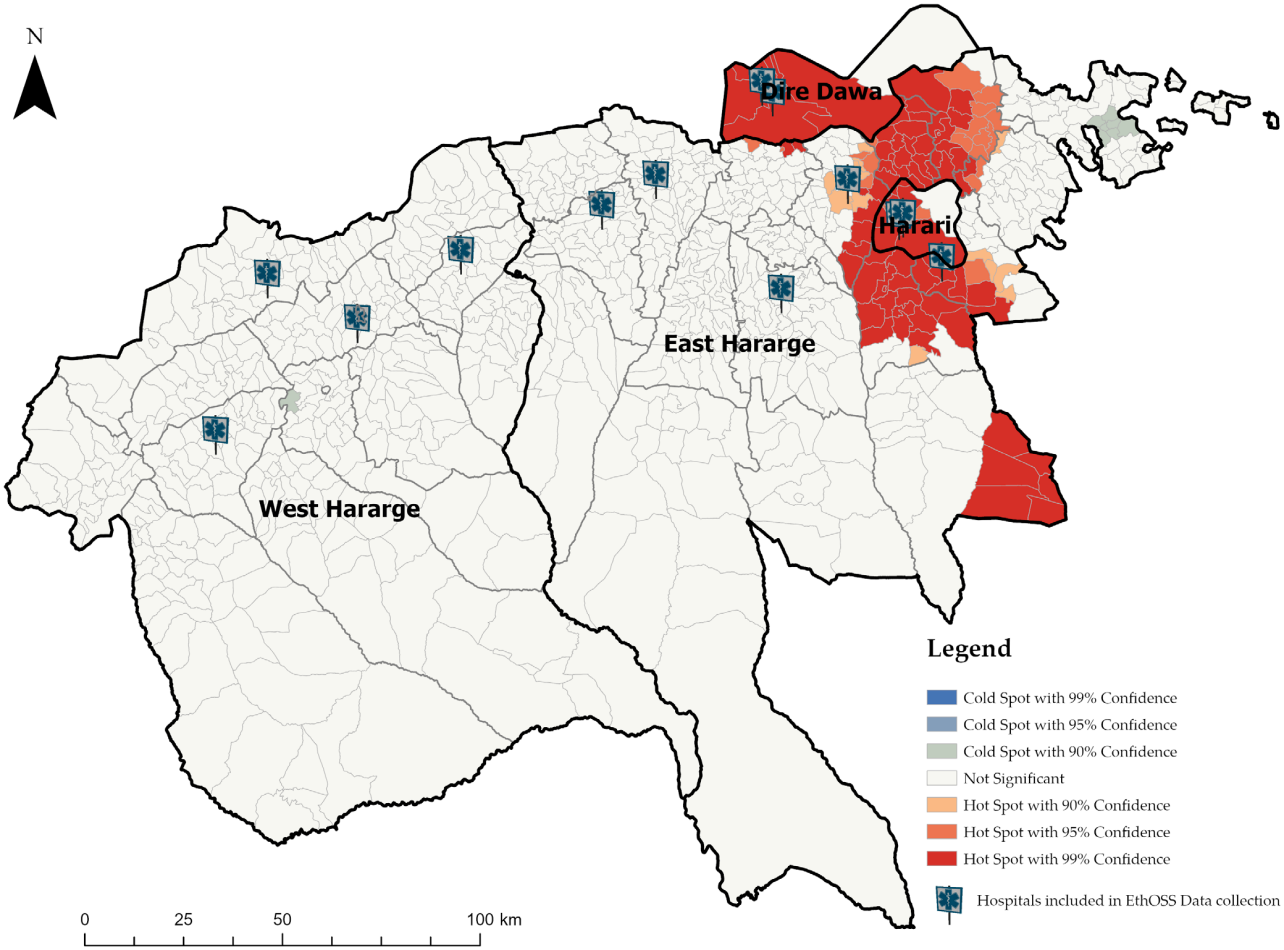
Hotspot and cold spot areas of severe maternal morbidity cases in eastern Ethiopia.

### 3.4 Geographically weighted regression

The closer a neighborhood is to an EmONC facility (*β* = 0.999, 95% confidence interval [CI] = 0.998–0.999) or any health facility (*β* = 0.996, 95% CI = 0.994–0.998), the higher the rate of severe maternal morbidities in the neighborhood. Similarly, a neighborhood whose closest facility is a comprehensive (*β* = 1.622, 95% CI = 1.326–1.985) or basic EmONC facility (*β* = 1.639, 95% CI = 1.278–2.104) had a higher severe maternal morbidity rate compared to a neighborhood whose closest facility is a non‐EmONC facility (Table 2). We believe this reflects care‐seeking in neighborhoods closer to a facility rather than risk, as discussed later in the discussion section.

**TABLE 2:** Frequency distribution of the explanatory variables and the results of the linear regression analysis.

Variable	Category	Frequency (%)	Estimate	*P*‐value
Age	(Mean (IQR))	25 (5)	1.012 (0.998–1.027)	0.102
ANC	Yes	541 (31%)	0.96 (0.836–1.102)	0.56
	No	1210 (69%)	—	
PPH	Yes	258 (15%)	1.134 (0.903–1.424)	0.279
	No	1517 (85%)	—	
APH	Yes	613 (35%)	1.018 (0.812–1.276)	0.879
	No	1162 (65%)	—	
Sepsis	Yes	184 (10%)	1.021 (0.791–1.318)	0.87
	No	1591 (90%)	—	
Anemia	Yes	631 (36%)	1.066 (0.865–1.313)	0.549
	No	1144 (64%)	—	
Gravidity	(Median (IQR))	3 (1–6)	1.004 (0.973–1.036)	0.785
Eclampsia	Yes	365 (21%)	1.116 (0.863–1.443)	0.404
	No	1410 (79%)	—	
Uterine rupture	Yes	47 (3%)	1.138 (0.786–1.647)	0.493
	No	1728 (97%)	—	
History of abortion	Yes	132 (7%)	1.03 (0.846–1.254)	0.768
	No	1643 (93%)	—	
Referred from another facility	Yes	981 (55%)	0.997 (0.871–1.141)	0.964
	No	794 (45%)	—	
Closest facility type	C‐EmONC	199 (11%)	1.622 (1.326–1.985)	<0.001*
	B‐EmONC	154 (9%)	1.639 (1.278–2.104)	<0.001*
	Non‐EmONC	1422 (80%)	—	
Time to any facility (min)	(Median (IQR))	48 (15–84)	0.996 (0.994–0.998)	<0.001*
Time to EmONC (min)	(Median (IQR))	94 (46–169)	0.999 (0.998–0.999)	0.023*
Place of birth	Facility	625 (43%)	—	
	En route	670 (46%)	0.906 (0.722–1.135)	0.389
	Home	152 (47%)	0.768 (0.588–1.004)	0.053
Hemoglobin (g/dL)	(Mean (SD))	9 (3)	0.999 (0.97–1.03)	0.97

Abbreviations: ANC, antenatal care; APH, antepartum hemorrhage; B‐EmONC, basic EmONC; C‐EmONC, comprehensive EmONC; EmONC, emergency obstetric and newborn care; IQR, interquartile range; PPH, post‐partum hemorrhage.

Figure 5 presents the results of the geographically weighted regression. This approach performs local regression analyses, examining the relationships between variables and severe maternal morbidity for each neighborhood. The resulting regression coefficients offer insights into the local magnitude and direction of these associations in each neighborhood, as well as their spatial variability across the study area.

**FIGURE 5:**
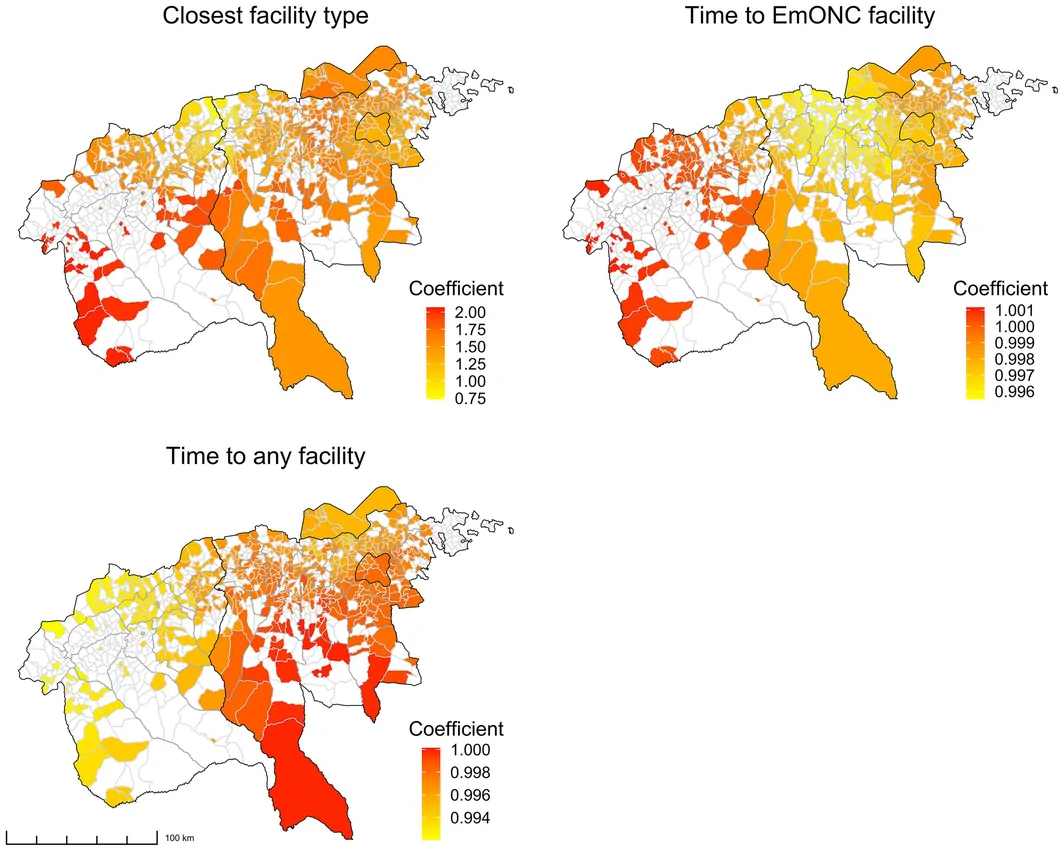
Spatial variation in the relationship between severe maternal morbidity rates and explanatory variables in eastern Ethiopia. (The colors in the figure represent the local regression coefficients, which indicate the magnitude of the association between the variables and severe maternal morbidity in each neighborhood. A higher coefficient suggests that the relationship between the variable and severe maternal morbidity is more pronounced in that particular location).

The geographically weighted regression analysis showed that the relationship between severe maternal morbidity rates and proximity to health facilities is not uniform across the study area. The association between severe maternal morbidity and the type of nearest facility was weaker in the central northern part of the region. Additionally, the relationship between severe maternal morbidity and travel time to the nearest facility, for both EmONC facilities and any health facility, varied between the eastern and western parts of the study area.

## 4 DISCUSSION

In this study, we employed geospatial analysis to identify geographic clusters of severe maternal morbidity and investigate the underlying factors contributing to this spatial distribution. We leveraged a statistical approach to analyze data collected through obstetric surveillance, addressing complex epidemiological and public health questions in a low‐resource setting. Using geocoding techniques, we were able to add map coordinates to the EthOSS data and perform spatial analysis. As a result, we uncovered the spatial pattern and relationships of severe maternal morbidity in eastern Ethiopia, which would not have been possible to detect.

We found a significant clustering pattern, indicating that severe maternal morbidities are not evenly distributed. We identified hotspot areas, which are statistically significant geographic clusters of high severe maternal morbidity rates, primarily near the larger cities of Dire Dawa and Harar. In contrast, coldspot areas with lower‐than‐expected severe maternal morbidity rates were found near the border with the Somali region and in the central parts of West Hararghe. It is worth noting that these severe maternal morbidity rates reflect where the women came from, not the location of the hospitals where they received care. Therefore, the hotspot and coldspot finding suggests that a high number of women with severe maternal morbidity visited hospitals from these bigger cities. This number is statistically significantly higher than what would be expected if the distribution were uniform across the study area, even after controlling for population density. This might mean that: (i) women from other areas never reach the hospital when complications arise and are, therefore, not counted; (ii) women from other areas simply die and therefore do not end up in the morbidity figures; (iii) women from the urban areas have complications more frequently and are, therefore, at higher risk; or (iv) women from the urban areas get “too much too soon,” including unnecessary intervention at the EmONC facilities such as the use of cesarean sections when not needed. We believe the first two scenarios are more likely the case as EthOSS is an institution‐based survey, which, unlike community‐based surveys, only included women who visited the hospital due to complications. Therefore, this group only represents women who decided to and are able to access hospital care. Hence, the hotspots in larger cities might reflect that more women visit hospitals in these areas than in other regions when complications arise due to higher awareness and better healthcare access. This highlights the inequality where only the urban population receives care when complications arise. It also suggests that women in rural and remote areas might fail to access health care when complications occur, leading to deaths at home or in lower‐level facilities. Based on data from a community‐based demographic and health survey, our national study found maternal and perinatal mortality cold spots in areas around Harari and Dire Dawa.^20^ Our qualitative investigation into the reasons for these inequalities suggests that the lower mortality in these regions might be attributed to higher health awareness among women and better healthcare access due to the urban setting.^30^ The observed geographic disparities in severe maternal morbidity highlight the need for targeted maternal health interventions in underserved areas of eastern Ethiopia. Policymakers and health planners could prioritize emergency obstetric services, strengthen referral, and improve transportation services in high‐risk and hard‐to‐reach communities. In addition, outreach and community‐based case identification in rural coldspot areas, where women might not reach hospitals when complications arise, is important. Embedding such approaches within existing health information systems could enhance early detection, improve access to emergency obstetric care, and reduce disparities in maternal health outcomes. Moreover, integrating geospatial surveillance into routine maternal health planning could support more equitable resource allocation by identifying areas with limited service utilization and potential under‐detection of severe maternal morbidity.

Our findings suggest that the spatial distribution of severe maternal morbidity is associated with the proximity and type of health facilities in a neighborhood. We observed that higher severe maternal morbidity rates were linked to the presence of a health facility in proximity. Further, neighborhoods whose closest facility was a comprehensive or basic EmONC center had higher severe maternal morbidity rates compared to those with a non‐EmONC facility nearby. These results support the notion that the severe maternal morbidity hotspots observed in the larger cities of Dire Dawa and Harari might be due to better healthcare access and greater awareness towards seeking medical care in these urban areas. These findings are consistent with the wide array of evidence suggesting that higher access to health care is associated with greater utilization of health services.^31^, ^32^ This pattern is also expected in countries such as Ethiopia, which are transitioning from the second to the third stage of the obstetric transition.^33^ In the second stage of the obstetric transition, the first and second delays—the delay in seeking and reaching care—are important factors.^29^, ^33^

This study successfully leveraged geocoding techniques to analyze existing survey data, enabling the description of spatial patterns and elucidation of factors associated with severe maternal morbidity in eastern Ethiopia. The EthOSS survey was not designed with geospatial analysis in mind, and consequently, no GPS data were collected. Using geocoding techniques to convert place names to map coordinates, we were able to conduct spatial analysis on the EthOSS data. This allowed us to harness the power of geospatial analysis, such as mapping and understanding inequalities, on a routine survey (i.e., one not originally set up for this purpose). This suggests the potential for utilizing routine health management information system data to conduct similar spatial analyses.^34^ However, the inherent methodological limitations of institution‐based surveys, which only capture women who decided to and were able to access hospital care, should be considered when interpreting findings from such data sources. Alternatively, data from existing community‐based surveys, such as the Health and Demographic Surveillance System,^35^ could be employed to investigate different aspects of maternal and child health disparities in Ethiopia. Such community‐level data could also serve to monitor progress and evaluate the impact of interventions within the population.

This study also had several limitations to consider when interpreting the data. First, this study is observational and based on an institution‐based survey, which could introduce selection bias, as it only captured women who accessed hospital care for severe complications. As a result, it likely underestimates the true burden of severe maternal morbidity by excluding women who did not reach hospitals due to geographic, financial, or sociocultural barriers, particularly in rural and remote areas. Additionally, the regression analyses excluded neighborhoods without reported severe maternal morbidity, as we lacked explanatory variable information for these areas because no women from those neighborhoods participated in the survey. Geocoding was performed at the kebele level using neighborhood centroids rather than precise GPS coordinates, which reduced spatial resolution and might have introduced positional uncertainty, particularly in larger or heterogeneous kebeles. This limitation might have affected the precision of hotspot detection and distance‐based analyses. The study also encountered challenges in mapping the reported addresses of women to predefined administrative divisions, including misspelled neighborhood names, multiple local names for the same area, inconsistent abbreviations, and the use of outdated administrative division names in areas with recent boundary changes. In addition, women whose residential addresses were unavailable or could not be matched to the neighborhood list in the locational data were excluded from the analysis. While the exclusion of these cases was necessary for the geospatial analysis, it might have introduced selection bias. Consequently, severe maternal morbidity in some areas might be under‐represented in the findings. To address these issues in future studies, we recommend placing greater emphasis on collecting comprehensive geographical information, such as improving spatial accuracy by collecting household‐level GPS data when possible or gathering details on the closest health facility or school to participants' residences, which could serve as a helpful reference point for accurately geocoding the data. Further, the lack of well‐functioning address systems, such as street names, postcodes, or zip codes, in Ethiopia made it more difficult to geolocate the women. This highlights the need to improve the national spatial data infrastructure.^36^ Finally, the cross‐sectional nature of the data precludes us from drawing causal inferences between the identified factors and the spatial distribution of severe maternal morbidity.

## 5 CONCLUSION

This study utilized geospatial analyses of obstetric surveillance data to identify geographic clusters and determinants of severe maternal morbidity in eastern Ethiopia. We found higher rates of severe maternal morbidity in the larger cities of Dire Dawa and Harar, where access to care during obstetric complications was more readily available. In contrast, rural and remote areas often lack healthcare access, with many women unable to reach facilities when complications arise and often die at home. These findings highlight significant urban–rural disparities in maternal health outcomes that might not have been detected without spatial analysis.

Integrating geospatial surveillance into routine maternal health planning could support more equitable resource allocation by identifying areas with limited service utilization and potential under‐detection of severe maternal morbidity. Spatial analyses can guide targeted outreach, referral strengthening, and community‐based case identification in rural coldspot areas where women might not reach hospitals when complications arise. Embedding such approaches within existing health information systems could enhance early detection, improve access to emergency obstetric care, and reduce urban–rural disparities in maternal health outcomes.

## AUTHOR CONTRIBUTIONS

Conception or design of the work: Sisay Mulugeta Alemu, Gerd Weitkamp, Marian Knight, Sagni Girma, Mohammed Yuya, Jelle Stekelenburg, Thomas van den Akker, Regien Biesma, and Abera Kenay Tura; data analysis and interpretation, Sisay Mulugeta Alemu, Abera Kenay Tura, Gerd Weitkamp, Jelle Stekelenburg, and Regien Biesma; drafting the article, Sisay Mulugeta Alemu; critical revision of the article, Gerd Weitkamp, Marian Knight, Sagni Girma, Mohammed Yuya, Jelle Stekelenburg, Thomas van den Akker, Regien Biesma, and Abera Kenay Tura. All authors have read and approved the manuscript prior to submission.

## FUNDING INFORMATION

The authors acknowledge funding from the University Medical Center Groningen (UMCG), Graduate School of Medical Science Ph.D. grant for SMA. The funder has no involvement in the design of the study, in the writing of the report, or in the decision to submit the paper for publication.

## CONFLICT OF INTEREST STATEMENT

The authors declare no conflicts of interest.

## ETHICS STATEMENT

The EthOSS project has been reviewed and approved by the Institutional Health Research Ethics Review Committee at Haramaya University's College of Health and Medical Sciences in Ethiopia (Ref No. IHRERC/024/2021), as well as the Oxford Tropical Research Ethics Committee at the University of Oxford (OxTREC Reference 530–21).

## DECLARATION OF GENERATIVE AI TECHNOLOGIES

During the preparation of this work, the authors used chatGPT to improve the manuscript's readability. After using this tool/service, authors reviewed and edited the content as needed and take full responsibility for the content of the publication.

## Data Availability

Research data are not shared.
